# Analysis of Rare Variants in the *C3* Gene in Patients with Age-Related Macular Degeneration

**DOI:** 10.1371/journal.pone.0094165

**Published:** 2014-04-15

**Authors:** Maheswara R. Duvvari, Codrut C. Paun, Gabriëlle H. S. Buitendijk, Nicole T. M. Saksens, Elena B. Volokhina, Tina Ristau, Frederieke E. Schoenmaker-Koller, Johannes P. H. van de Ven, Joannes M. M. Groenewoud, Lambertus P. W. J. van den Heuvel, Albert Hofman, Sascha Fauser, André G. Uitterlinden, Caroline C. W. Klaver, Carel B. Hoyng, Eiko K. de Jong, Anneke I. den Hollander

**Affiliations:** 1 Department of Ophthalmology, Radboud University Nijmegen Medical Centre, Nijmegen, the Netherlands; 2 Radboud Institute for Molecular Life Sciences, Radboud University Nijmegen Medical Centre, Nijmegen, the Netherlands; 3 Department of Ophthalmology, Erasmus Medical Centre, Rotterdam, the Netherlands; 4 Department of Epidemiology, Erasmus Medical Centre, Rotterdam, the Netherlands; 5 Department of Pediatric Nephrology, Radboud University Nijmegen Medical Centre, Nijmegen, the Netherlands; 6 Department of Vitreoretinal Surgery, Department of Ophthalmology, University Hospital of Cologne, Cologne, Germany; 7 Department for Health and Evidence, Radboud University Nijmegen Medical Centre, Nijmegen, the Netherlands; 8 Netherlands Consortium for Healthy Aging, Netherlands Genomics Initiative, the Hague, the Netherlands; 9 Department of Internal Medicine, Erasmus Medical Centre, Rotterdam, the Netherlands; 10 Department of Human Genetics, Radboud University Nijmegen Medical Centre, Nijmegen, the Netherlands; Justus-Liebig-University Giessen, Germany

## Abstract

Age-related macular degeneration (AMD) is a progressive retinal disorder affecting over 33 million people worldwide. Genome-wide association studies (GWASs) for AMD identified common variants at 19 loci accounting for 15–65% of the heritability and it has been hypothesized that the missing heritability may be attributed to rare variants with large effect sizes. Common variants in the complement component 3 (*C3*) gene have been associated with AMD and recently a rare *C3* variant (Lys155Gln) was identified which exerts a large effect on AMD susceptibility independent of the common variants. To explore whether additional rare variants in the *C3* gene are associated with AMD, we sequenced all coding exons in 84 unrelated AMD cases. Subsequently, we genotyped all identified variants in 1474 AMD cases and 2258 controls. Additionally, because of the known genetic overlap between AMD and atypical hemolytic uremic syndrome (aHUS), we genotyped two recurrent aHUS-associated *C3* mutations in the entire cohort. Overall, we identified three rare variants (Lys65Gln (*P* = 0.04), Arg735Trp (OR = 17.4, 95% CI = 2.2–136; *P* = 0.0003), and Ser1619Arg (OR = 5.2, 95% CI = 1.0–25; *P* = 0.05) at the *C3* locus that are associated with AMD in our EUGENDA cohort. However, the Arg735Trp and Ser1619Arg variants were not found to be associated with AMD in the Rotterdam Study. The Lys65Gln variant was only identified in patients from Nijmegen, the Netherlands, and thus may represent a region-specific AMD risk variant.

## Introduction

Age-related macular degeneration (AMD, MIM 603075) is a retinal disorder that causes progressive visual impairment in individuals aged over 50 years [1]. AMD primarily affects the macula, the central region of the retina, eventually leading to loss of central and sharp vision. It has been estimated that more than 33 million people suffer from vision loss due to AMD worldwide [2]. AMD is a multifactorial disease caused by a combination of genetic and environmental factors. GWASs identified common variants at 19 loci that influence disease susceptibility, accounting for 15–65% of the heritability [3], [4].

It has been hypothesized that the remaining genetic fraction influencing the risk for development of AMD, the so-called missing heritability, may be explained by rare, highly penetrant variants [4]. Simulation studies suggested that common variants are insufficient to account for disease burden in densely affected AMD families and that rare penetrant variants would offer a likely explanation [5]. In addition, a meta-analysis of AMD GWASs suggested that each of the 19 loci may harbour several independent variants associated to AMD susceptibility [3]. The rare variant hypothesis is supported by the identification of rare missense mutations in the fibulin-5 (*FBLN5*) gene and the hemicentin-1 (*HMCN1*) gene in AMD patients [6], [7]. In addition, rare, highly penetrant variants in the genes encoding complement factor H (*CFH*), complement factor I (*CFI*), complement component 3 (*C3*) and complement component 9 (*C9*) have recently been found to be associated with AMD [8]–[12].

Genetic studies have identified an important role for the complement cascade in the pathogenesis of AMD [13]. Interestingly, recent studies suggested a genetic overlap between AMD and atypical hemolytic uremic syndrome (aHUS), a life-threatening renal disease caused by chronic, uncontrolled activation of the complement system. It has been reported that 4–10% of aHUS patients carry mutations in the *C3* gene [14]. Moreover, disease-causing mutations previously identified in aHUS patients, such as Arg1210Cys in *CFH*, Gly119Arg in *CFI* and Lys155Gln in *C3* were found to confer a high risk of developing AMD [8]–[12]. However, the precise nature of this genetic overlap between two clinically distinct phenotypes remains unknown.

In this study, we explored the role of rare variants in the *C3* gene in the pathogenesis of AMD. We performed a two-stage analysis to identify rare variants in the *C3* gene. First, sequence analysis was carried out in a discovery set of 84 AMD cases from the EUGENDA cohort, and subsequently the frequencies of these variants were determined in replication sets from EUGENDA and from the Rotterdam Study consisting of 1474 AMD cases and 2258 controls. In addition, two recurrent aHUS-associated *C3* mutations were genotyped in the entire AMD case-control cohort [15], [16].

## Results

To investigate the involvement of rare variants in the *C3* gene in AMD, the exons and flanking introns of *C3* were sequenced in a discovery cohort of 84 AMD cases (Table 1) from the Nijmegen area, the Netherlands. Sequencing identified three rare variants (MAF<1%; Lys155Gln/rs147859257, Arg735Trp/rs117793540 and Ser1619Arg/rs2230210) and two common variants (MAF≥1%; Arg102Gly/rs2230199 and Pro314Leu/rs1047286) (Table 2). None of the rare variants were found in 192 ethnicity-matched and age-matched controls. The Lys155Gln variant, which has recently been associated with AMD [10]–[12], was found in five cases, while variants Arg735Trp and Ser1619Arg were found in one case each. Bioinformatic algorithms SIFT and PolyPhen predicted the variants Arg102Gly, Lys155Gln, and Pro314Leu not to be damaging whereas Arg735Trp and Ser1619Arg were predicted to be damaging to the protein function (Table 2).

**Table 1:** Demographics of studied subjects.

Variables	EUGENDA cohort		Rotterdam Study	
	Cases	Controls	Cases	Controls
Controls (n)		1246		1012
Intermediate AMD (n)	173		636	
Advanced AMD (n)	545		120	
Mean age (±SD)	76±8	70±5.9	80±6.4	77±6.5
Gender				
Male	271	532	354	497
Female	447	714	402	515

EUGENDA: a multicenter database comprising participants from Germany and the Netherlands.

**Table 2:** *C3* variants identified by sequence analysis of 84 AMD cases.

SNP ID	Sequence variants		Genotypes		Prediction algorithms	
	Nucleotide change	Amino acid change	Mm	mm	SIFT	PolyPhen2
rs2230199	c.304 C>G	Arg102Gly	34	11	Tolerated (0.5)	Tolerated (0)
rs1047286	c.941 C>T	Pro314Leu	30	10	Tolerated (0.1)	Tolerated (0.2)
rs147859257	c.463 A>C	Lys155Gln	5	0	Tolerated (0.2)	Benign (0.1)
rs117793540	c.2203 C>T	Arg735Trp	1	0	Deleterious (0)	Damaging (1)
rs2230210	c.4855 A>C	Ser1619Arg	1	0	Deleterious (0)	Damaging (0.8)

Major and minor allele indicated in capital and lower case respectively.

Reference sequence of *C3* (NM_000064) gene.

SIFT: Sorting Intolerant from Tolerant (Intolerance ≤0.05).

PolyPhen2: Polymorphism Phenotyping (score 0→1).

Next, the frequencies of the rare variants Arg735Trp and Ser1619Arg, as well as the common variants Arg102Gly and Pro314Leu were determined, in replication cohorts from EUGENDA and from the Rotterdam Study, consisting of 1474 AMD cases and 2258 controls of European ancestry (Table 3). In addition, two recurrent aHUS mutations (Lys65Gln and Arg161Trp) were included in the analysis (Table 3). The common variants Arg102Gly (OR = 1.2 [95% CI 1.1–1.4]; *P* = 0.001) and Pro314Leu (OR = 1.2 [95% CI 1.0–1.4]; *P* = 0.005) were significantly associated with AMD. In the EUGENDA cohort, the rare variant Arg735Trp was found heterozygously in 8 and homozygously in one out of 718 AMD cases and heterozygously in one out of 1246 controls (OR = 17.4 [95% CI 2.2–136]; *P* = 0.0003). However, in the Rotterdam Study the rare variant Arg735Trp was found heterozygously in 1 out of 785 AMD cases and heterozygously in 2 out of 1048 controls, and was thus not associated with the disease. Rare variant Ser1619Arg was found heterozygously in six out of 718 AMD cases and heterozygously in two out of 1244 controls in the EUGENDA cohort (OR = 5.2 [95% CI .1.0–25]; *P* = 0.05). In the Rotterdam Study the rare variant Ser1619Arg was found heterozygously in 6 out of 835 AMD cases and heterozygously in 11 out of 1279 controls, and was thus not associated with AMD. The aHUS mutation Lys65Gln was found heterozygously in three out of 717 AMD cases and was not observed in 1246 controls (*P* = 0.05) in the EUGENDA cohort, but was not identified in the Rotterdam Study. Arg161Trp was found heterozygously in two out of 644 AMD cases and was not observed in 1142 controls (*P* = 0.13) in the EUGENDA cohort. In the Rotterdam Study the aHUS mutation Arg161Trp was observed heterozygously in one out of 320 AMD cases and heterozygously in one out of 483 controls (*P* = 1.0), and was not significantly associated with AMD in the EUGENDA cohort nor in the Rotterdam Study.

**Table 3:** Genotyping of *C3* variants in EUGENDA and Rotterdam samples.

SNP ID	Amino acid change	EUGENDA cohort				Rotterdam Study				Combined cohorts (EUGENDA and Rotterdam)			
		MAF (%)		OR (95% CI)	p-value (2-sided)	MAF (%)		OR (95% CI)	p-value (2-sided)	MAF (%)		OR (95% CI)	p-value (2-sided)
		Controls	Cases			Controls	Cases			Controls	Cases		
Common variants													
Rs2230199	Arg102Gly	20.2	24.4	1.2 (1.1-1.4)	0.001	21.2	24.0	1.1 (1.0-1.3)	0.04	20.6	24.2	1.2 (1.1-1.3)	0.0002
Rs1047286	Pro314Leu	19.5	23.5	1.2 (1.0–1.4)	0.005	20.6	23.1	1.1 (0.9–1.3)	0.06	20.0	23.3	1.2 (1.0–1.3)	0.001
Rare variants													
	Lys65Gln	0	0.20	ND	0.04	0	0	NA	1.00	0	0.14	NA	0.05
	Arg161Trp	0	0.15	ND	0.13	0.10	0.15	1.5 (0.09–24)	1.00	0.03	0.15	5.0 (0.5–48)	0.14
rs117793540	Arg735Trp	0.04	0.69	17.4 (2.2–136)	0.0003	0.09	0.06	0.7 (0.06–8.4)	1.00	0.06	0.36	6.1 (1.7–21.6)	0.003
Rs2230210	Ser1619Arg	0.08	0.41	5.2 (1.0–25)	0.05	0.43	0.35	0.8 (0.30–2.2)	0.80	0.25	0.38	1.5 (0.6–3.2)	0.31

Major and minor allele indicated in capital and lower case respectively, MAF: Minor allele frequency, ND: OR could not be determined, NA: Not applicable.

EUGENDA: a multicenter database comprising participants from Germany and the Netherlands.

To determine whether the identified variants conferred disease risk independent of the two known common *C3* variants (Arg102Gly/rs2230199 and Pro314Leu/rs1047286), a conditional logistic regression analysis was performed (Table 4). After conditioning on Arg102Gly/rs2230199, Arg735Trp remained associated with disease risk in the EUGENDA cohort (OR = 22.1, 95% CI = 2.8–173; *P* = 0.003). Similarly, after conditioning on Pro314Leu/rs1047286, Arg735Trp still showed association with disease risk in the EUGENDA cohort (OR = 22.0, 95% CI = 2.8–172; *P* = 0.003). In addition, Arg735Trp was significantly associated with disease risk in the EUGENDA cohort (OR = 22.1, 95% CI = 2.8–173; *P* = 0.003) after conditioning on both variants (Arg102Gly and Pro314Leu). Independent association with AMD could not be assessed for Lys65Gln and Ser1619Arg because too few data points were available to perform a reliable conditional analysis.

**Table 4:** Conditional analysis of Arg735Trp for SNPs Arg102Gly/rs2230199 and Pro314Leu/rs1047286.

Condition	EUGENDA cohort		Combined cohorts (EUGENDA and Rotterdam)	
	OR (95%CI)	p-value	OR (95%CI)	p-value
Arg102Gly/rs2230199	22.1 (2.8–173)	0.003	6.2 (1.7–22.5)	0.005
Pro314Leu/rs1047286	22.0 (2.8–172)	0.003	6.2 (1.7–22.4)	0.005
Arg102Gly & Pro314Leu	22.1 (2.8–173)	0.003	6.2 (1.7–22.5)	0.005

EUGENDA: a multicenter database comprising participants from the Cologne area, Germany and the Nijmegen area, the Netherlands.

## Discussion

In this study, three rare variants (Lys65Gln, Arg735Trp and Ser1619Arg) in the *C3* gene were shown to be associated with AMD disease risk in our EUGENDA cohort. However, the Arg735Trp and Ser1619Arg variants were not found to be associated with AMD in the Rotterdam Study. The Arg735Trp and Ser1619Arg variants were also not associated with AMD in a recent study that analyzed the *C3* gene in 2,493 AMD cases and controls [10]. The Lys65Gln variant was only identified in AMD patients from the Nijmegen area, while it was not found in the Rotterdam Study, nor in a cohort from Boston [10]. The Lys65Gln variant may therefore represent a region-specific AMD risk variant, being confined to the east of the Netherlands, which is confirmed by the occurrence of Lys65Gln in Dutch aHUS patients [16]. A fourth variant, Arg161Trp, was found in three AMD cases and in one control; therefore it cannot be ruled out that the variant is in fact associated with AMD but a larger sample size would be required to detect a significant association.

The complement system plays an important role in the pathogenesis of AMD [8]–[12]. The *C3* gene encodes the complement component 3 protein, a central component of the complement cascade that plays a crucial role in clearance of pathogens and immune complexes. All three branches of the complement system, the classical pathway, the mannose-binding lectin pathway and the alternative pathway, converge at C3. At this stage, activation of C3 results in an amplification loop that ultimately forms a cytolytic membrane attack complex which targets pathogens. The system is tightly controlled by molecules like CFH and CFI. If the complement cascade is improperly regulated, host cells may also be subjected to complement attack, resulting in tissue damage and a spectrum of complement-mediated diseases [17].

The rare variants reported in this study have previously been described for their association with aHUS, a chronic renal disorder caused by uncontrolled activation of the complement system, often leading to renal failure. Functional experiments have been performed in this context to determine the effect of the amino acid substitutions on C3 function. The Lys65Gln variant resides in the macroglobulin (MG1) domain of C3 and was shown to cause decreased binding to CFH [16]. Although not significantly associated in our study, the Arg161Trp variant was shown to cause hyperactive C3 convertase due to increased binding to factor B (CFB), and reduced binding to CFH and membrane cofactor protein (MCP; CD46), resulting in higher complement activity [15], [16], [18].

Recombinant protein studies have shown that the Arg735Trp variant performs normal in binding and cleavage assays [14]. However, since the Arg735Trp residue is located in the anaphylotoxin (C3a) domain of C3, it is not likely to show an effect in the performed assays that evaluated binding and activation of the C3b fragment. Recent studies in animal models suggest that C3a anaphylotoxin has specific functions in the retina and retinal pigment epithelium, but it remains unknown how an amino acid substitution in C3a may contribute to the development of AMD [19], [20].

The Lys65Gln variant associated with AMD in this study has previously been associated with aHUS, further supporting the genetic overlap between AMD and aHUS. To rule out any renal pathology, the medical histories of AMD patients who carried this variant were evaluated, but no signs of aHUS or other renal pathologies were reported in these patients. Thus, although aHUS and AMD may overlap genetically, in this study no clinical overlap was shown, suggesting that compounding (genetic or environmental) factors contribute to a particular clinical phenotype. Such a notion is further supported by a study that showed that some aHUS patients carry multiple mutations in the complement factor genes [21]. In order to understand the shared associations, cross-phenotype studies are warranted to unravel the mechanisms common and unique to aHUS and AMD. This will lead to more rational approaches to diagnosis and therapy by targeting these specific molecular targets.

The identification of rare penetrant AMD-associated variants may have relevance for diagnostic, predictive and therapeutic purposes, although the exact interpretation may remain a challenge. Recent studies have identified several highly penetrant rare variants (Lys155Gln in *C3*, Gly119Arg in *CFI*, Pro167Ser in *C9* and Arg1210Cys in *CFH*), to be associated with AMD [8]–[12]. However, not all associations hold true among different populations. An example of this is the Arg1210Cys variant in *CFH* which was strongly associated in a North American AMD cohort [8] but not in an Icelandic AMD cohort [11] (Table 5). Genetic drift, founder effects or differences in genetic make-up that could compensate for a rare disruptive variant may underlie this phenomenon. In addition, the possibility that environmental effects could mask or enhance the penetrance of certain alleles between populations may also exist. To date, it remains unclear how these observations impact the predictive value of finding such variants in individuals. In contrast, variants that were proven to be functionally impaired and that are associated with AMD in several populations, such as the Lys155Gln variant in *C3*, have a much stronger predictive value.

**Table 5:** Rare variants in AMD studies.

Gene	Variant	Study (Population)	Functional implication	Conclusions
Present study				
C3	Lys65Gln	NL	CFH binding ↓ [16]	Associated with AMD, not present in other populations, proven functionality, reported in aHUS [16]
	Arg161Trp	NL	CFH binding ↓, CFB binding ↑ [15], [16], [18]	Not associated with AMD, proven functionality, reported in aHUS [15], [16], [18]
	Arg735Trp	NL	Normal binding [14]	Not associated with AMD, normal functionality, reported in aHUS [14]
	Ser1619Arg	NL	NA	Not associated with AMD, not proven functionality, not found in aHUS
Other studies				
C3	Lys155Gln	ISL, NL, GER, USA [10], [11], [12]	CFH binding ↓ [18]	Associated with AMD, replicated in several populations, proven functionality, reported in aHUS [18]
CFI	Gly119Arg	NL, USA [9]	CFI activity ↓ [9]	Associated with AMD, replicated in different populations, proven functionality, reported in aHUS [9], [30]
C9	Pro167Ser	FRA, USA [10]	NA	Associated with AMD, replicated in different populations [10]
CFH	Arg1210Cys	USA [8]	C3b, heparin and endothelial cells binding ↓ [31]	Associated with AMD, not present in other populations, proven functionality, reported in aHUS [31]

NL: The Netherlands, ISL: Iceland, GER: Germany, USA: United states of America, FRA: France.

NA: Not applicable.

In conclusion, we report a rare variant (Lys65Gln) at the *C3* locus in patients with AMD, while an association with two other variants (Arg735Trp and Ser1619Arg) was not confirmed in other cohorts. This study further supports that rare variants contribute to the genetic variance of AMD, which may have implications for predictive testing and personalized medicine in AMD [10].

## Materials and Methods

### Cases and controls

Cases and controls included in this study were selected from the population-based Rotterdam Study, Rotterdam, The Netherlands and from the European Genetic Database (EUGENDA), a multicenter database comprising participants from the Cologne area, Germany and the Nijmegen area, the Netherlands. All participants of this study underwent extensive retinal imaging which has been described in detail elsewhere [22], [23]. In short, AMD staging was performed by grading of stereo fundus photographs according to the standard protocol of the Rotterdam grading center and Cologne Image Reading Center (CIRCL). All subjects were classified on the basis of the eye with the more severe diagnosis. Cases were aged ≥50 years of age and AMD was classified by the presence of at least 15 intermediate (63–124 µm) or at least one large drusen (≥125 µm) or geographic atrophy or choroidal neovascularisation secondary to AMD. Control subjects were aged ≥65 years of age and did not have AMD (none or only small, hard drusen or only pigmentary abnormalities or less than 10 small drusen and pigmentary abnormalities). Written informed consent was obtained from all participants. The EUGENDA study was approved by the local research ethics committee, Commissie Mensgebonden Onderzoek Regio Arnhem-Nijmegen, the Netherlands, and Ethics Committee of the University Hospital Cologne, Germany. The Rotterdam study was approved by the institutional review board (Medical Ethics Committee) of the Erasmus Medical Center and by the review board of The Netherlands Ministry of Health, Welfare and Sports. The study was performed in accordance with the tenets of the Declaration of Helsinki.

### Sequencing

Sanger sequencing of the *C3* (NM_000064) gene was performed in the discovery set, consisting of 84 AMD patients selected from the EUGENDA database. Primers were designed for all 41 coding exons and intron-exon boundaries by Primer3 software (Supplementary Material, Table S1). Polymerase chain reaction (PCR) was performed, and PCR amplicons were sequenced using an automated sequencer (BigDye Terminator, version 3, 3730 DNA analyzer; Applied Biosystems). Sequences were assembled and analysed using ContigExpress (Vector NTI Advance, Version 11.0, Life Technologies). Each newly identified variant was confirmed by a second independent PCR and bidirectional Sanger sequencing. The predicted effects of identified missense variants were examined using Polymorphism Phenotyping (PolyPhen) [24] and Sorting Intolerant from Tolerant (SIFT) [25].

### Genotyping in the EUGENDA cohort

Variants (Lys65Gln, Arg102Gly, Arg161Trp, Pro314Leu, and Arg735Trp) were genotyped in the EUGENDA cohort using competitive allele-specific PCR assays (KASPar SNP Genotyping System, KBiosciences). KASPar genotyping was performed according to the manufacturer's protocol in a volume of 4 µl containing 10 ng of genomic DNA, 2.5 µl of 2× reaction mix, and 0.069 µl of assay. Thermal cycling conditions included a pre-incubation step at 94°C for 15 min, 20 cycles of 94°C for 10 s, 57°C for 5 s, 72°C for 10 s, followed by 23 cycles of 94°C for 10 s, 57°C for 20 s, 72°C for 40 s. Plates were analyzed on a 7900 Fast Real-Time PCR system (Applied Biosystems). The Ser1619Arg variant was genotyped in the EUGENDA cohort by Amplification Refractory Mutation System (ARMS) [26] PCR (Supplementary Material, Table S2). PCR reactions were performed in a volume of 12.5 µl using 20 ng genomic DNA, 1× buffer, 2.5 mM MgCl_2_, 1 mM deoxyribonucleotide triphosphates, 0.2 µM of each primer, and 0.5 U Taq DNA polymerase (Invitrogen, Life technologies). Thermal cycle conditions included a pre-incubation step at 95°C for 5 min, 16 cycles of 95°C for 30 s, 69°C for 30 s, 72°C for 45 s, followed by 25 cycles of 95°C for 30 s, 67°C for 30 s, 72°C for 45 s. PCR amplicons were analysed by agarose gel electrophoresis. Each newly identified variant was confirmed by a second independent PCR and bidirectional Sanger sequencing.

### Exome sequencing, exome chip analysis and genotyping in the Rotterdam Study

The occurrence of the *C3* variants Lys65Gln, Arg161Trp, Arg735Trp, and Ser1619Arg in the Rotterdam Study (RS) was retrieved from exome chip and exome sequencing data. For exome sequencing purposes, genomic DNA of RS participants was prepared from blood and fragmented into 200–400 bp fragments using Covaris Adaptive Focused Acoustics (AFA) shearing according to the manufacturer's instructions (Covaris, Inc., Woburn, MA). Illumina TruSeq DNA Library preparation (Illumina, Inc., San Diego, CA) was performed on a Caliper Sciclone NGS workstation (Caliper Life Sciences, Hopkinton, MA), followed by exome capture using the Nimblegen SeqCap EZ V2 kit (Roche Nimblegen, Inc., Madison, WI). This capture targets 44 Mb of exonic regions covering 30,246 coding genes, 329,028 exons and 710 miRNAs. Paired-end 2 ×100 sequencing was performed on Illumina HiSeq2000 sequencer using Illumina TruSeq V3 chemistry. Downstream analyses included demultiplexing (CASAVA software, Illumina), alignment using the burrows-wheeler alignment tool, followed by data processing and filtering with Picard, SAMtools and the Genome Analysis Tool-Kit [27], [28]. Finally, variant detection was performed using GATK's Unified Genotyper. For exome chip analysis, DNA samples of the Rotterdam study were included in the joint calling experiment of the Cohorts for Heart and Aging Research in Genomic Epidemiology (CHARGE) Consortium [29]. In short, DNA from the study participants was processed on the HumanExome BeadChip v1.0 (Illumina, Inc, San Diego, CA) querying 247,870 variable sites using standard protocols at seven genotyping centers. Each center genotyped a common set of 96 HapMap samples to be utilized for quality control and determination of batch effects. Raw datafiles for all samples were transferred to a central location and assembled into a single joint calling (Illumina GenomeStudio v2011.1 software and GenTrain 2.0 clustering algorithm). Call rates >99% were used for both study samples as for HapMap controls to define genotype clusters. Finally 8994 variants were excluded for further analyses. Common variants (Arg102Gly and Pro314Leu) were genotyped in the RS cohort using Taqman assays (Applied Biosystems, Foster city, California, USA).

### Statistical analysis

A Fisher's exact test was performed to assess the association between each variant and AMD, and also to check for Hardy-Weinberg equilibrium. P-values were calculated two-sided, and values of <0.05 were considered as statistically significant. Logistic regression analysis was performed to check for genotype interactions, to estimate the odds ratios (OR) with 95% confidence intervals (CI) and to adjust for age. Conditional analysis was performed to identify secondary association signals at the *C3* locus by accounting for two known AMD SNPs. Either Arg102Gly/rs2230199 or Pro314Leu/rs1047286 was added independently and combined to the regression model as a covariate to test the effect of the variant of interest. Data were analyzed using SPSS software, version 18.0.

## Supporting Information

Table S1 **List of** ***C3*** **gene sequencing primers.**
(DOC)

Table S2 **Genotyping probes: List of Amplification Refractory Mutation System (ARMS) and kaspar primers.**
(DOC)
