# Supplementary material for: Analysis of Rare Variants in the C3 Gene in Patients with Age-Related Macular Degeneration
Source: PLoS One. 2014 Apr 15;9(4):e94165. doi: 10.1371/journal.pone.0094165 (PMC3988049; doi:10.1371/journal.pone.0094165)
Supplement: Table S1 — List of C3 gene sequencing primers. (DOC) [file pone.0094165.s001.doc]

**SUPPLEMENTARY MATERIAL**

Table S1, List of *C3* gene sequencing primers

| Primers | Sequence (5’- 3’) | Product (bp) |
| --- | --- | --- |
| Exon 1F | TGCTCACTCCTCCCCATC | 199 |
| Exon 1R | AAATGTCTGCTTCCACCCC |  |
| Exon 2F | GGCGTCTCACATCCGTG | 333 |
| Exon 2R | GAAGACAGAAGGGGAGGGG |  |
| Exon 3F | AGATCCGGAAGCTGGACC | 444 |
| Exon 3R | TTGCCTCTCCTAAGCCTGTG |  |
| Exon 4F | AGCGGGTACCTCTTCATCC | 300 |
| Exon 4R | CCTTCCGGTGTGTCTTTCTC |  |
| Exon 5-6F | TAGACACTGTGCACAGAGAAT | 516 |
| Exon 5-6R | TTTCTCTGTAGGCTCCACTAT |  |
| Exon 7F | AAGATCCGAGCCTACTATGAA | 311 |
| Exon 7R | GTCCCCCACCTGGTCTTCACC |  |
| Exon 8-9F | GGAGATCCCATTCTCCAGG | 455 |
| Exon 8-9R | TTTCTCTTCTGACCTGGTCTCC |  |
| Exon 10-11F | GGAGGTCTAATCCTGAGGGG | 500 |
| Exon 10-11R | GACCCCACTGTGCAAACAC |  |
| Exon 12F | CAGGTCTCAGGGATTCGG | 349 |
| Exon 12R | GAAGGAGTCCCAGGGGTG |  |
| Exon 13F | GAGGCCAAGATCCGCTACTAC | 546 |
| Exon 13R | GACAGTTGAGAGACAGAGAGGG |  |
| Exon 14F | AACCTTTCTGTCTTTCCACTC | 422 |
| Exon 14R | CATTCCCATCTTCAGCTTCAA |  |
| Exon 15-16F | CACAGGTGCATATGTGGGG | 629 |
| Exon 15-16R | TCCCCTCCTCCCTCTCTG |  |
| Exon 17F | GGGGAAGTCCTCCCTGG | 360 |
| Exon 17R | TCCCTCCTCAGACAGGAGTC |  |
| Exon 18-19F | TTTCACCATGTTAGCTAGGCT | 662 |
| Exon 18-19R | AATGAGATGACACTCAGACAC |  |
| Exon 20-21F | CTAAGAGCTGAGACCCAGGAG | 585 |
| Exon 20-21R | GAAGACCAGGAGCCCTCTC |  |

Table S1, List of *C3* gene sequencing primers

| Primers | Sequence (5’- 3’) | Product (bp) |
| --- | --- | --- |
| Exon 22-23F | TGCTGACCATCTGTGTGTCTG | 421 |
| Exon 22-23R | AATGAGATGGAATTTGGCTCC |  |
| Exon 24F | AACCCTTTTCACGCCACC | 343 |
| Exon 24R | GGATCTTAGGGGAGGGATGC |  |
| Exon 25F | TGAGTCCTTCCCTTTTTAAGG | 406 |
| Exon 25R | TCCGTGCTTAAGGATGCTTAA |  |
| Exon 26F | GGTTGACATGGCAGTCTCTG | 290 |
| Exon 26R | CTCTCGTGTTCATCCTGCG |  |
| Exon 27F | GATGACTGCCATGTGTGGAC | 241 |
| Exon 27R | CTGTGCTCTGCATCGGG |  |
| Exon 28F | AAGTGCTGCTCGAATGATCC | 297 |
| Exon 28R | CAGTATCTCCCGCCCTGAAC |  |
| Exon 29F | CTCTTTCTGAGCTTTCTCTGA | 386 |
| Exon 29R | AACTGATTCTCAACTCCACTG |  |
| Exon 30-31F | GATTCTAGCCACTTTCCCAGG | 495 |
| Exon 30-31R | AGAGGAGATGGTCCCTCTGG |  |
| Exon 32-33F | GACCATCTCCTCTTGTCCCC | 432 |
| Exon 32-33R | ACTTGGAAAGTACTGAATATCATGG |  |
| Exon 34-35F | TCCTTGTCCAGGAACAGACC | 424 |
| Exon 34-35R | CCAGCCAGATAGAGGTCAGG |  |
| Exon 36F | CAAGACAATGCTGGACTCCC | 244 |
| Exon 36R | CCCCACAATTCATATATACCTGG |  |
| Exon 37-38F | TCTTTGGAGGGAGGCCC | 504 |
| Exon 37-38R | TGACAACCACACCTACCACC |  |
| Exon 39-40F | TGCCCCTCATGGTCAAC | 428 |
| Exon 39-40R | ACAATGGTGTGGGCGTG |  |
| Exon 41F | CCACACCATTGTCACGCC | 280 |
| Exon 41R | GGCAAAGAACTCCAGACACG |  |
