# Supplementary material for: Analysis of Rare Variants in the C3 Gene in Patients with Age-Related Macular Degeneration
Source: PLoS One. 2014 Apr 15;9(4):e94165. doi: 10.1371/journal.pone.0094165 (PMC3988049; doi:10.1371/journal.pone.0094165)
Supplement: Table S2 — Genotyping probes: List of Amplification Refractory Mutation System (ARMS) and kaspar primers. (DOC) [file pone.0094165.s002.doc]

**SUPPLEMENTARY MATERIAL**

Table S2, Genotyping probes:

Ser1619Arg variant, Amplification Refractory Mutation System (ARMS) primer list

| Primers | Sequence (5’- 3’) | Product (bp) |
| --- | --- | --- |
| Wild type-Forward | CCTGACCTGCCATTCTTCCCTCCAGCCTTA | 298 |
| Mutant type-Forward | CCTGACCTGCCATTCTTCCCTCCAGCCTTC |  |
| Reverse | GGTTTCAAGTAGGATGGAGCTGAGCTGCAGGTG |  |

| List of kaspar primers |  |  |  |  |  |
| --- | --- | --- | --- | --- | --- |
| Variants | Allele X Primer | Allele Y Primer | Common Primer | Allele X | Allele Y |
| Lys65Gln | GTCCACGACTTCCCAGGCA | GTCCACGACTTCCCAGGCC | GTCTTCTCACTGGACAGCACTAGTT | A | C |
| Arg102Gly/rs2230199 | CACGGTCACGAACTTGTTGCC | CACGGTCACGAACTTGTTGCG | GCCAACAGGGAGTTCAAGTCAGAAA | C | G |
| Arg161Trp | CAATGTTGACCATGACCGTCCG | CAATGTTGACCATGACCGTCCA | TTCACCGTCAACCACAAGCTGCTA | C | T |
| Pro314Leu/rs1047286 | CCACCAGGTCTTCTGCTCGGA | CACCAGGTCTTCTGCTCGGG | TACTGCTGGACGGGGTGCAGAA | A | G |
| Arg735Trp/rs117793540 | GCAACTACATCACAGAGCTGC | CTGCAACTACATCACAGAGCTGT | TGGCCAGGCCCAGGTGGCT | C | T |
|  |  |  |  |  |  |
